# Analysis of Genotypes and Phenotypes in Chinese Patients With Tuberous Sclerosis Complex Harboring Novel Variants of TSC1 and TSC2 Genes

**DOI:** 10.1155/ijog/6963280

**Published:** 2025-05-08

**Authors:** Jian Chen, Hairui Sun, Ling Han, Xiaoyan Gu, Xiaoyan Hao, Yuwei Fu, Zongjie Weng, Yi Xiong, Baomin Liu, Hongjia Zhang, Yihua He, Hong Li

**Affiliations:** ^1^Department of Echocardiography, The First Affiliated Hospital of Shenzhen University, Shenzhen Second People's Hospital, Shenzhen, China; ^2^Maternal-Fetal Consultation Center of Congenital Heart Disease, Department of Echocardiography, Beijing An Zhen Hospital, Capital Medical University, Beijing, China; ^3^Department of Ultrasonography, Fujian Maternal and Child Hospital, Fuzhou, Fujian, China; ^4^Department of Ultrasonography, Shenzhen People's Hospital, Shenzhen, China; ^5^Department of Ultrasonography, The Second Affiliated Hospital, Medical College of Xi'an Jiao Tong University, Xi'an, China; ^6^Department of Cardiac Surgery, Beijing An Zhen Hospital Affiliated to Capital Medical University, Beijing, China; ^7^Department of Ultrasound, The Fourth Clinical Medical College of Guangzhou University of Chinese Medicine (Shenzhen Traditional Chinese Medicine Hospital), Shenzhen, China

**Keywords:** diagnosis, point variant, TSC1, TSC2, tuberous sclerosis complex

## Abstract

**Background:** This study aimed to assess the pathogenicity of newly identified tuberous sclerosis Complex 1 (TSC1) and TSC2 variants, contributing definitive evidence for the diagnosis of TSC.

**Methods:** A total of 103 TSC patients underwent TSC genetic testing using standardized protocols, and genetic testing was extended to their respective families. Analysis of genetic testing results considered clinical phenotype and gene pathogenicity based on the 2012 revision of the International Society of TSC.

**Results:** Among participants, 12 exhibited previously unreported variants of TSC1 or TSC2 gene absent in relevant databases. All 12 clinically diagnosed TSC patients presented typical phenotypes, such as brain lesions and skin changes. Notably, there were 2 variants of *TSC1* gene and 10 variants of *TSC2* gene, encompassing 8 frameshift variants, 2 nonsense variants, and 2 missense variants.

**Conclusions:** This study broadens the spectrum of variants of *TSC1* and *TSC2* genes, reaffirming the clinical diagnosis of patients through genetic testing.

## 1. Introduction

Tuberous sclerosis complex (TSC) is an inherited disorder impacting multiple organ systems, extensively documented by Bourneville in 1880 [1]. Manifesting predominantly in childhood or during fetal development, TSC exhibits pathological changes characterized by hamartoma formation and tissue constitution deficiency, influencing diverse organs, such as the brain, heart, skin, kidneys, and liver [2]. The spectrum of manifestations varies, with epilepsy and mental retardation representing severe symptoms [3]. As an autosomal dominant disorder, TSC has a global estimated incidence ranging from 1 in 5000 to 10,000 live births, with a male-to-female ratio of 2:1 [4–8]. The International TSC Association revised clinical diagnostic criteria in 2012, acknowledging pathogenic gene variants of TSC1 and TSC2 as independent diagnostic standards [9, 10].

The *TSC1* gene, located on chromosome 9q34, spans 55 kb and encodes hamartin [11], while the *TSC2* gene, on chromosome 16p13.3, spans 40 kb and encodes tuberin [12]. Hamartin and tuberin form the TSC protein complex, which is crucial in regulating the mammalian target of rapamycin signaling pathway [1]. At present, around 85% of TSC patients exhibit definitive variants in either *TSC1* or *TSC2* genes [13–15]. Genetic testing serves as a vital complement to clinical diagnosis, particularly for patients with less apparent clinical phenotypes, with significant applications in prenatal TSC diagnosis [16, 17].

This study aimed to validate clinical diagnoses by testing the TSC target gene in patients clinically diagnosed or highly suspected of TSC. Simultaneously, the pathogenicity of the variants was analyzed, and the relationship between genotype and phenotype was assessed for selected variants.

## 2. Materials and Methods

### 2.1. Patients

In collaboration with TSC China Alliance, a cohort comprising 103 unrelated TSC patients and their families underwent TSC1 and TSC2 genetic testing between October 2015 and June 2022. Following the TSC Diagnostic Criteria revised by the International TSC Association in 2012 [9], all patients were clinically diagnosed with TSC. The age range at the time of diagnosis spanned from 31 weeks of gestation to 43 years, with the smallest patient being a 31-week gestational age fetus. Comprehensive medical records and family histories were meticulously examined.

### 2.2. Informed Consent

Prior to genetic testing, all patients provided informed consent, signifying their awareness and agreement to participate in the study. The consent encompassed the collection of specific samples, such as amniotic fluid or umbilical cord blood and peripheral blood. Families declining informed consent were excluded from the study. This research received ethical approval from the Medical Ethics Committee of Beijing An Zhen Hospital Affiliated with Capital Medical University (Beijing, China) (approval no. L201610) and was performed in compliance with the Declaration of Helsinki.

### 2.3. Management of Patients

Peripheral blood samples were systematically collected from both TSC patients and their respective family members. Target gene detection and subsequent clinical follow-up were conducted as part of the study protocol. In the case of the two fetuses within the cohort, umbilical cord blood puncture was performed to procure fetal gene samples, given their more advanced gestational age. All samples, including those from peripheral blood and umbilical cord blood puncture, were collected using 2-mL ethylenediaminetetraacetic acid anticoagulant tubes. Each participant provided two tubes, ensuring a sufficient volume for analysis. The collected samples were promptly refrigerated for preservation at 4°C and subsequently dispatched for comprehensive genetic analysis.

### 2.4. Detection of TSC Variants

DNA extraction from umbilical cord blood and peripheral blood was carried out using the QIAamp DNA Blood Kit (Qiagen, Hilden, Germany). All exons of *TSC1* and *TSC2* genes were amplified utilizing multiplexed PCR (hema9600, Zhuhai, China), and subsequent sequencing of amplicons was conducted on the HiSeq 2500 platform (Illumina, San Diego, California, USA) in accordance with established protocols.

#### 2.4.1. Primary PCR Reaction

PCR primers and running conditions for each exon were obtained from previous research [18]. Each exon underwent PCR amplification with a total sample volume of 25 *μ*L, consisting of 12.5 *μ*L HiFi Taq multiplex (2×), 4 *μ*L primer mix (approximately 0.2–2 *μ*M each), and 8.5 *μ*L DNA (30 ng), topped up with ddH_2_O. The PCR process initiated with a denaturing step at 95°C for 3 min, followed by 18 cycles of denaturing at 95°C for 15 s, annealing at 65°C for 30 s, and extension at 72°C for 45 s. The reaction was concluded with a final cycle at 72°C for 30 s, and the samples were subsequently stored at 4°C. Purification of the product was performed using AMPure XP (1.2×) following standard procedures, with elution in ddH_2_O (12 *μ*L). The supernatant was retained for subsequent reactions.

#### 2.4.2. Secondary PCR Reaction

The secondary PCR reaction, with a total sample volume of 25 *μ*L, included 12.5 *μ*L HiFi Taq multiplex (2×), 10.5 *μ*L recovery products from the primary reaction, 1 *μ*L TruSeq GF, and 1 *μ*L TruSeq Index (10 *μ*M each). PCR amplification commenced with a denaturing step at 98°C for 3 s, followed by 18 cycles of denaturing at 98°C for 10 s, annealing at 65°C for 30 s, and extension at 72°C for 30 s. A final cycle at 72°C for 2 min concluded the reaction, and samples were stored at 4°C. Purification of the product was carried out using 1× AMPure XP according to standard procedures, with elution in 25 *μ*L ddH_2_O.

#### 2.4.3. Sample Sequencing

Subsequent to sample collection, amplicons were sequenced using the HiSeq 2500 platform (Illumina) in accordance with standardized protocols. Sequencing reads were aligned to the human reference genome (hg19) through BWA, and variations were identified utilizing SAMtools and Pindel. Custom scripts were employed for further filtering, considering qualitative metrics and information from databases, such as dbSNP, OMIM, and SwissVar. The remaining variations underwent prediction of physiological outcomes using PROVEAN and SIFT. All identified deleterious variants were subsequently validated through Sanger sequencing.

#### 2.4.4. Multiplex Ligation-Dependent Probe Amplification (MLPA)

When no suspected pathogenic variants are identified using the single-nucleotide assay, we proceed with MLPA to detect potential copy number variations (CNVs).

### 2.5. Interpretation of the Genetic Report

Clinicians and genetic counselors conducted a comprehensive analysis, correlating biological information, clinical phenotype, symptoms, and treatment status within the patient's pedigree. Utilizing this amalgamation of data, a genetic diagnosis was meticulously formulated [19, 20]. This multidimensional approach ensures a robust and thorough interpretation of the genetic report.

### 2.6. Limitations

The sample underwent high-throughput sequencing of the coding regions of TSC1 and TSC2 using a multiplex PCR. Subsequently, next-generation sequencing was performed to verify the sites of potentially harmful variants. It should be noted that this test does not cover genetic structural variations, such as heterozygous deletions of large fragments, duplications, and inversion rearrangements. Additionally, large fragment heterogeneity, synthetic insertion variants, and variants located in noncoding regions are not included.

## 3. Results

### 3.1. TSC Targets Gene Variants

In this study, we identified a total of 87 variants in the TSC genes across 103 patients. This included 16 variants in *TSC1*, of which 14 had been previously reported and 2 were novel. Additionally, 71 variants were found in *TSC2*, comprising 61 previously reported variants and 10 novel variants. The remaining 17 patients tested negative for any TSC variants (Table S1).

### 3.2. Genetic Testing Results of Patients With Novel TSC Variants

**Figure 1:**
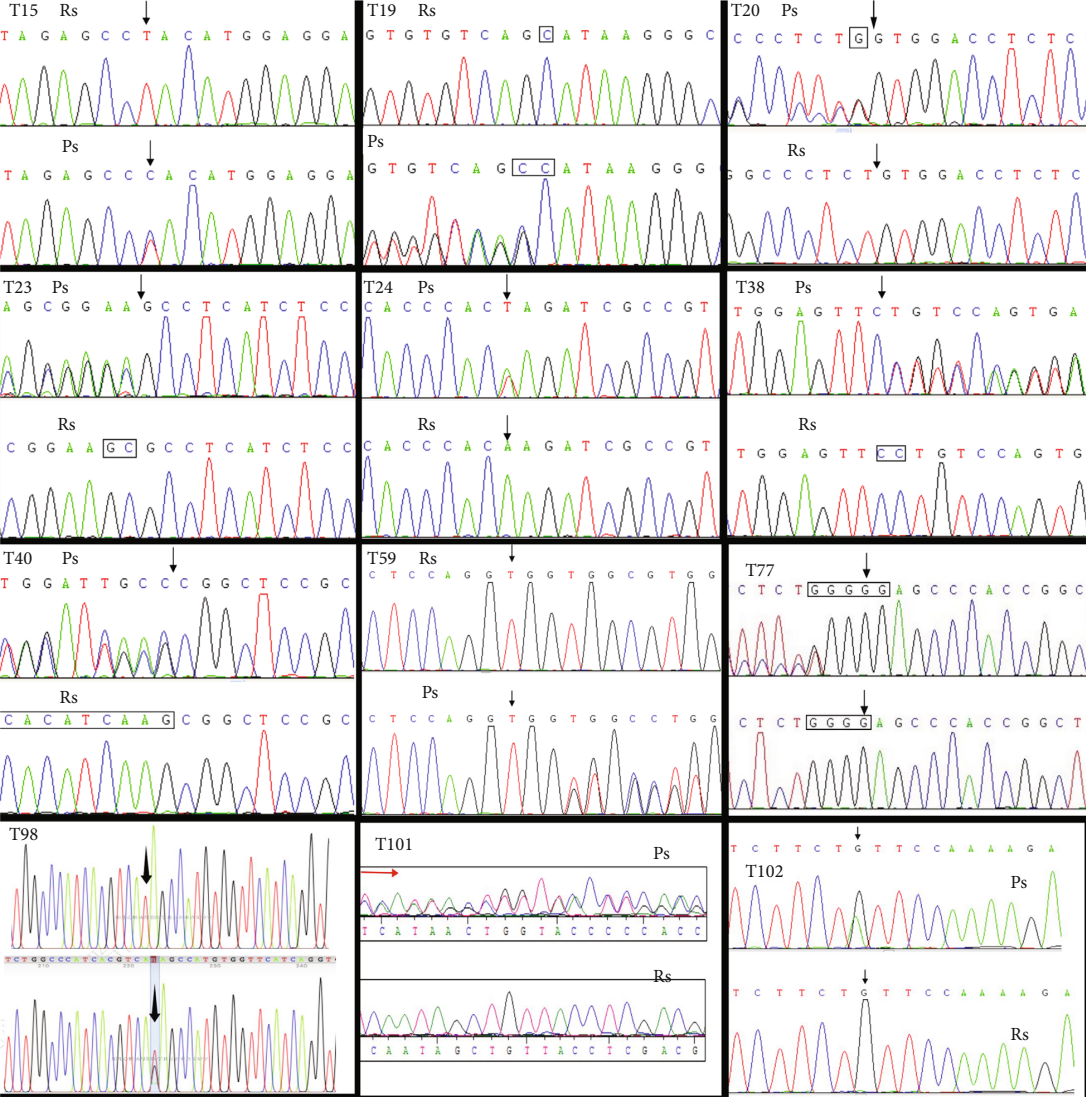
Molecular analysis identified 12 novel TSC gene variants by Sanger sequencing: T15, TSC2 c.853T>C; T19, TSC1 c.892_893insC; T20, TSC2 c.4113_4114insG; T23, TSC2 c.5384_5385delGC; T24, TSC2 c.4630A>T; T38, TSC2 c.2538del C; T40, TSC2 c.5227_5244delCGGCTCCGCCACATCAAG; T59, TSC2 c.1064_1065insG; T77, TSC2 c.910_911insG; T98, TSC2 c.T2678G; T101, TSC2 c.557delT; and T102, TSC1 c. 2623C>T. Ps, patient sequence; Rs, reference sequence. Black arrow: location of the variant site; red arrow: location of frameshift variant segment.

Among the 103 patients, 12 were found to harbor distinct novel *TSC* variants, all of whom had been clinically diagnosed with TSC. Variant analysis of the exons and exon/intron junctions of both *TSC1* and *TSC2* genes identified 2 novel variants in *TSC1* and 10 in *TSC2*. The specific types of variants included 8 frameshift variants, 2 nonsense variants, and 2 missense variants (Figure 1 and Table 1). These variants were absent in major databases, such as the Human Genome Mutation Database (HGMD) and Leiden Open Variation Database, as well as control databases (e.g., the 1000 Genomes Project, the NHLBI GO Exome Sequencing Project [ESP], and the Exome Aggregation Consortium [ExAC]).

### 3.3. Characteristics of Patients With Novel TSC Variants

Detailed clinical characteristics of 12 patients with novel TSC variants are presented in Table 2. Cardiac rhabdomyoma emerged as the most prevalent lesion, which was diagnosed in 10 of 12 patients. Additionally, a higher incidence of brain lesions was found, with 9 patients displaying subependymal nodules. Notably, brain lesions were identified as early as the prenatal period, around 31 weeks of gestation. Epilepsy and infantile spasms were frequent, affecting 4 (67%) of 6 children, while the remaining 6 were fetuses, posing challenges in monitoring fetal epilepsy/spasms. The second most common lesion was hypomelanotic macule, which was found in 6 of 12 patients, with the youngest patient exhibiting this feature at 6 months. Notably, 3 of 12 patients inherited TSC variants from their families, showcasing different clinical phenotypes compared with the previous generation (Table 1).

**Figure 2:**
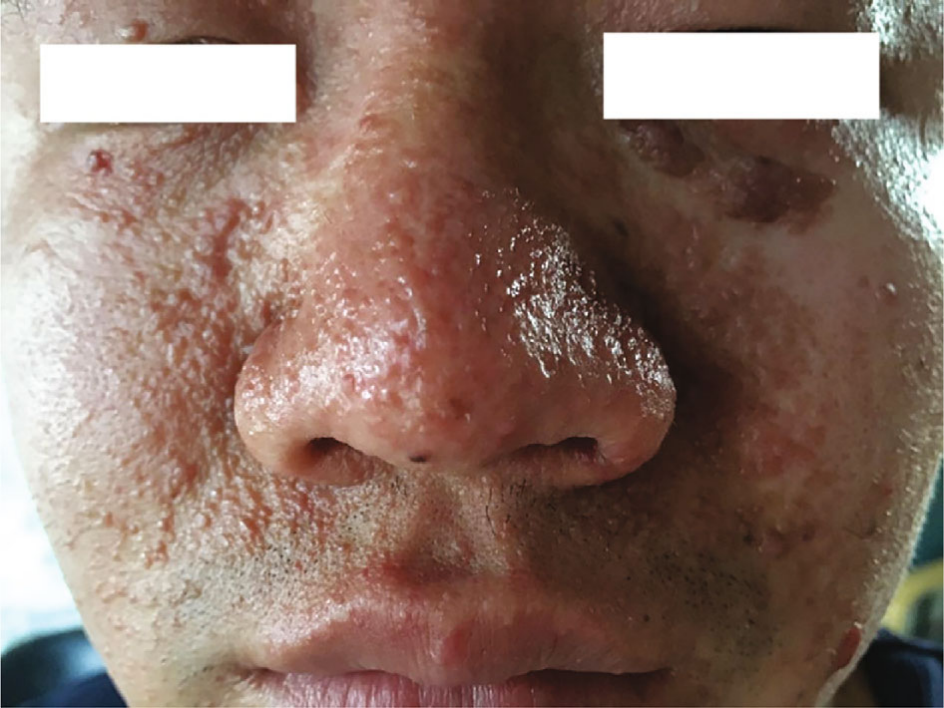
T15 is a TSC family genetic case. The figure shows the proband's father with multiple angiofibromas on his face.

The clinical manifestations among family members of the three patients with the hereditary form of TSC varied. In the T15 family, the proband's son exhibited typical cutaneous manifestations of TSC, including multiple facial angiofibromas and shagreen patches (Figure 2), while the proband's grandchild was diagnosed with multiple cardiac rhabdomyomas via prenatal ultrasound. However, in this family, we identified a suspected pathogenic missense mutation, but did not detect a definitive pathogenic mutation or CNV. In the T19 family, the proband's mother presented with multiple facial fibromas and shagreen patches. In the T102 family, the proband's mother had a mild clinical presentation, characterized by multiple hypomelanotic macules on her back.

## 4. Discussion

In this study, variants in the TSC1 gene were identified in 16 of 103 families (16%), while TSC2 variants were detected in 70 families (67%). The remaining 17 families (17%) did not show any TSC gene variants. These findings are broadly consistent with previous reports [19]. Because most missense variants are benign or of uncertain clinical significance, only two missense variants (T15 and T98) were identified in our cohort. Pathogenicity was evaluated according to the 2015 guidelines of the American College of Medical Genetics and Genomics and the Association for Molecular Pathology (ACMG/AMP; see Table 1 and Table S1). Based on this assessment, both missense variants were classified as potentially pathogenic.

TSC, an autosomal dominant genetic disorder, exhibits systemic involvement and phenotypic diversity affecting various organs, including the skin, central nervous system, heart, eyes, and kidneys. TSC patients' prognosis is primarily associated with epilepsy, manifesting in 80% of affected children, and mental retardation. Early diagnosis and intervention have shown effectiveness in symptom relief [6, 21–23].

In the current study, 12 patients presented varying degrees of brain lesions, with particular emphasis on two fetal patients. The presence of brain lesions in fetuses underscores the likelihood that brain variations represent a prevalent early stage pathology in TSC. Furthermore, the majority of patients with brain lesions exhibited neuropsychiatric disorders and epilepsy. Detecting and addressing early stage brain lesions becomes pivotal for effective management [24]. Notably, studies demonstrated that patients with TSC2 variants experience earlier onset and more severe clinical manifestations compared with those carrying TSC1 variants [25, 26]. However, the treatment of fetal TSC remains a topic of debate due to potential influences on fetal brain development.

Skin changes, particularly hypomelanotic macules, were common in the present study, affecting 6 out of 12 patients. These skin lesions, often less conspicuous in the early stages, are frequently overlooked by patients, leading to delayed diagnosis and treatment. Numerous patients in this cohort were not diagnosed with TSC until they sought medical attention at our center due to the onset of epilepsy symptoms.

**Figure 3:**
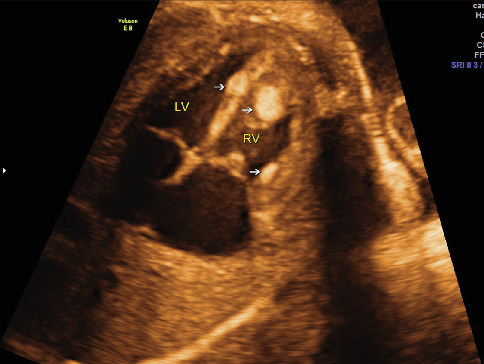
Fetal echocardiography showed multiple masses in the right ventricular and ventricular septum (as indicated by the arrow).

Cardiac rhabdomyoma emerged as the most prevalent specific lesion in this study, aligning with the typical symptomatology of TSC, especially multiple cardiac rhabdomyomas (Figure 3) [27–29]. Studies suggested that almost 99% of patients with multiple cardiac rhabdomyomas are eventually diagnosed with TSC [30–33]. In the present study, 10 out of 12 patients were diagnosed with multiple cardiac rhabdomyomas, both clinically and genetically confirming the presence of TSC.

With advancements in technology, the diagnosis of TSC has been revolutionized, primarily concentrating on alterations in the *TSC1* and *TSC2* genes. The International TSC Congress in 2012 recommended changes in these genes as an independent diagnostic criterion [9]. To date, the HGMD has recorded over 300 variants of the *TSC1* gene and 1000 variants of the *TSC2* gene. Nearly 500 of these variants have been classified as definitively pathogenic in the NCBI ClinVar database. Frameshift variants, characterized by a significant alteration in nucleotide order leading to marked abnormalities in amino acid synthesis, are the most prevalent among these pathogenic variants [34].

In the present study, 8 out of 12 novel TSC variants identified were frameshift variants, which is consistent with previously reported findings [18]. These variants, including TSC1 c.892_893insC, TSC2 c.4113_4114insG, TSC2 c.5384_5385delGC, TSC2 c.2538delC, TSC2 c.5227_5244delCGGCTCCGCCACATCAAG, TSC2 c.1064_1065insG, TSC2 c.910_911insG, and TSC2 c.557delT, are detailed in Table 1, highlighting specific changes in related proteins.

Most missense mutations are benign mutations or mutations of unknown significance. In our study, we identified two novel missense variants in *TSC2*: c.2678T>G (p.I893R), located at an infrequent site in Exon 24, and c.853T>C (p.Y285H), located in Exon 10. The first variant TSC2 c.2678T>G (p.I893R) could predict the replacement of isoleucine at Position 893 of the coding protein with arginine, reflecting a significant difference in physicochemical properties between the two amino acid residues. Multiple prediction tools, including SIFT, PolyPhen-2, and MutationTaster, unanimously indicated that the variant is likely harmful. Notably, this variant was not present in common benign variant databases (e.g., ExAC, 1KGP, and ESP6500), indicating its uncommon nature. The second variant, *TSC2* c.853T>C (p.Y285H), results in a substitution of tyrosine (Y) with histidine (H) at Position 285. Protein structure predictions using PROVEAN software suggest that this variant may be harmful. Given that this family exhibits typical clinical manifestations of TSC and the mutation site follows the geneticist's cosegregation pattern, we conclude that the c.853T>C variant has a high likelihood of pathogenicity.

Furthermore, this study detected two nonsense variants: TSC2 c.4630A>T (p.K1544X,264) and TSC1 c. 2623C>T (p.Q875X). These variants introduce a premature stop codon, leading to early termination of the encoded protein. This comprehensive gene variant analysis sheds light on the diverse molecular alterations contributing to TSC pathogenesis, emphasizing the importance of understanding the underlying genetic landscape to develop diagnostic and therapeutic strategies.

As reported previously, approximately two-thirds of TSC patients are sporadic, while the remaining one-third have familial cases [4, 35, 36]. Consistently, only 3 out of 10 patients in this study had hereditary TSC cases. The genetic variants identified in these three families included a missense variant (*TSC2* c.853T>C), a nonsense variant (*TSC1* c.2623C>T), and a frameshift variant (*TSC1* c.892_893insC). Of particular note is the observation that the previous generation of these familial cases exhibited no serious clinical symptoms, especially lacking apparent neuropsychiatric cognitive disorders compared to the subsequent generation. This discrepancy in clinical manifestations across generations emphasizes the complex interplay of genetic factors and highlights the intricate nature of TSC inheritance. Understanding the genetic characteristics within familial cases provides valuable insights for genetic counseling and contributes to a more comprehensive comprehension of TSC pathogenesis.

While this study has conducted a thorough genetic analysis of the 12 recently identified variants associated with TSC, it is imperative to acknowledge several limitations for a nuanced interpretation of the findings. The investigation predominantly centered on genetic analysis, shedding light on the probable high pathogenicity of the identified TSC variants. Nevertheless, to bolster the robustness of our conclusions, further research involving functional tests is essential. Experimental assays can unveil the true impact of these variants on protein function, contributing to a more comprehensive understanding of their role in TSC pathogenesis. The study's reliance on a relatively small sample size may constrain the generalizability of the results, as the analysis encompassed only 12 new TSC variants, limiting the statistical power to establish significant correlations with distinct clinical features. Addressing these limitations requires larger cohorts that can unravel potential associations obscured in smaller samples. Additionally, owing to the inherent constraints of the sample size, the present study did not find any apparent correlation between a specific TSC variant type and distinct clinical features, emphasizing the need for a more extensive dataset covering diverse variant types and a broader range of clinical manifestations.

## 5. Conclusions

This study conducted a comprehensive analysis of TSC target genes within 103 families associated with TSC, uncovering 12 novel variants. The identification of these variants represents a significant contribution to the existing knowledge, expanding the spectrum of genetic variations associated with *TSC1* and *TSC2* genes implicated in the development of TSC. By assessing the genetic landscape of TSC, this study enriched an understanding of the molecular underpinnings of this complex disorder. The novel variants not only contribute to the growing repository of genetic data but also hold promise for advancing diagnostic capabilities and potential therapeutic interventions for individuals affected by TSC. In essence, this research extended the boundaries of our knowledge regarding variants in *TSC1* and *TSC2* genes, underscoring the ongoing efforts to unravel the intricacies of TSC pathogenesis. As the scientific community continues to assess the genetic intricacies of TSC, the discoveries made in this study pave the way for future investigations aimed at elucidating the broader genetic landscape and fostering advancements in the clinical management of TSC.

## Figures and Tables

**Table 1:** Characteristics of 12 novel TSC gene variants which have not been reported.

**Patients no.**	**Location**	**Nucleotide alteration**	**Amino acid alteration**	**Type of variant**	**De novo*/*hereditary**	**NM-No**	**Supporting evidence** ^**a**^	**Pathogenicity** ^**b**^
T15	TSC2 (Exon 10)	c.853T>C	p.Y285H	Missense	Hereditary	NM_000548.3	PS1, PM2, PP1, PP3	II
T19	TSC1 (Exon 9)	c.892_893insC	p.A298AfsX2	Frameshift	Hereditary	NM_000368.4	PVS1, PS1, PM2, PP1	I
T20	TSC2 (Exon 34)	c.4113_4114insG	p.V1371Gfs42	Frameshift	De novo	NM_000548.3	PVS1, PS2, PM2, PP1	I
T23	TSC2 (Exon 41)	c.5384_5385delGC	p.K1794Kfs∗	Frameshift	De novo	NM_000548.3	PVS1, PS2, PM2, PP1	I
T24	TSC2 (Exon 36)	c.4630A>T	p.K1544X,264	Nonsense	De novo	NM_000548.3	PVS1, PS2, PM2, PP1	I
T38	TSC2 (Exon 22)	c.2538delC	p.F846Ffs∗48	Frameshift	De novo	NM_000548.3	PVS1, PM2, PP1, PP4	I
T40	TSC2 (Exon 41)	c.5227_5244delCGGCTC CGCCACATCAAG	p.R1743_K1748del	In-frame Deletion	De novo	NM_000548.3	PVS1, PS2, PM2, PP1, PP4	I
T59	TSC2 (Exon 11)	c.1064_1065insG	p.(V355Vfs∗32)	Frameshift	De novo	NM_000548.3	PVS1, PS2, PM2, PP1, PP4	I
T77	TSC2 (Exon 10)	c.910_911insG	p.W304Wfs∗34	Frameshift	De novo	NM_000548.3	PVS1, PS2, PM2, PM4, PP1	I
T98	TSC2 (Exon 24)	c. 2678T>G	p.I893R	Missense	De novo	NM_000548.3	PS2, PM2, PP3, PP4	II
T101	TSC2 (Exon 6)	c.557delT	p.F186Sfs∗16	Frameshift	De novo	NM_000548.3	PVS1, PS2, PM2, PM4, PP4	I
T102	TSC1 (Exon 20)	c. 2623C>T	p.Q875X	Nonsense	Hereditary	NM_001162427	PVS1, PM2, PM4, PP4	I

Abbreviation: TSC, tuberous sclerosis complex.

^a^The classification of genetic pathogenicity is based on the following reference: Richards et al. standards and guidelines for the interpretation of sequence variants: a joint consensus recommendation of the American College of Medical Genetics and Genomics and the Association for Molecular Pathology. Genet Med, 2015.17(5): p. 405-24.

^b^Classification of pathogenicity of variants: I, pathogenic; II, likely pathogenic, III, uncertain significance, IV, likely benign, and V, benign.

**Table 2:** Main clinical symptoms of 12 patients with novel TSC gene variants.

**No.**	**Age of diagnosis**	**Gender**	**HM**	**MA**	**Epilepsy/IS**	**Brain lesions**	**Neuropsychiatric disorders**	**CR**	**Renal AML**	**Other**
T15	43 years	Male	+	+	−	CD	−	+	+	SP
T19	3.5 years	Male	+	+	+	CD, SEN	+	−	−	−
T20	4 years	Male	+	+	−	SEN	−	+	+	RAP
T23	6 years	Female	+	+	+	CD, SEN	−	+	−	−
T24	1 year	Male	+	+	+	CD, SEN	+	−	−	−
T38	32 weeks^a^	−	−	−	ND	CD, SEN	ND	+	−	−
T40	2 years	Male	+	+	+	CD, SEN	+	+	+	−
T59	31 weeks^a^	−	−	−	ND	CD, SEN	ND	+	−	−
T77	26 weeks^a^	−	−	−	ND	ND	ND	+	−	−
T98	24 weeks^a^	−	−	−	ND	ND	ND	+	−	−
T101	31 weeks^a^	−	−	−	ND	ND	ND	+	+	−
T102	33 weeks^a^	−	−	−	ND	SEN	ND	+	−	−

Abbreviations: AML, angiomyolipomas; CD, cortical dysplasia; CR, cardiac rhabdomyoma; HM, hypopigmented macules; IS, infantile spasm; MA, multiple angiofibroma; ND, not determined; RAP, retinal achromic patch; SEN, subependymal nodules; SP, shagreen patch.

^a^Fetal gestational age (the parents of all six abnormal fetuses chose to terminate their pregnancies, and all of these fetuses had multiple or single cardiac rhabdomyomas on prenatal fetal echocardiography, and some fetuses also had multiple subependymal nodules on fetal brain MRI findings).

## Data Availability

The datasets generated and analyzed during the current study are available from the corresponding authors on reasonable request.

## Nomenclature

AML: angiomyolipomas
CD: cortical dysplasia
CR: cardiac rhabdomyoma
CNV: copy number variant
F: familial
HM: hypopigmented macules
IS: infantile spasm
MA: multiple angiofibroma
N: nonreported
ND: not determined
Ps: patient sequence;
RAP: retinal achromic patch
Rs: reference sequence
S: sporadic
SEN: subependymal nodules
SP: shagreen patch
TSC: tuberous sclerosis complex
W: weeks
