# Supplementary material for: Analysis of Genotypes and Phenotypes in Chinese Patients With Tuberous Sclerosis Complex Harboring Novel Variants of TSC1 and TSC2 Genes
Source: Int J Genomics. 2025 May 8;2025:6963280. doi: 10.1155/ijog/6963280 (PMC12081158; doi:10.1155/ijog/6963280)
Supplement: Supporting Information — Additional supporting information can be found online in the Supporting Information section. Table S1 Characteristics of 103 TSC gene variants. [file 6963280.f1.docx]

**Supplementary Table 1 Characteristics of 103 TSC gene variants.**

| Patients no. | Location | Nucleotide alteration | Type of variant | Pathogenicity |
| --- | --- | --- | --- | --- |
| T1 | TSC2(Exon30) | c.3412C>T | nonsense | pathogenic |
| T2 | TSC2(Exon36) | c.4578del C | frameshift | pathogenic |
| T3 | TSC1(Exon15) | c.1525C>T | nonsense | likely pathogenic |
| T4 | TSC2(Exon12) | c.1139T>C | missense | likely pathogenic |
| T5 | — | — | — | — |
| T6 | TSC2(Exon30) | c.3696dup T | frameshift | pathogenic |
| T7 | TSC2(Exon30) | c.3599G>C | missense | likely pathogenic |
| T8 | TSC2(Exon1-2) | Loss of heterozygosity | CNV | pathogenic |
| T9 | — | — | — | — |
| T10 | TSC1(Exon18) | c.2359G>T | nonsense | likely pathogenic |
| T11 | TSC1(Exon21) | c.2251C>T | nonsense | likely pathogenic |
| T12 | — | — | — | — |
| T13 | TSC1(Exon18) | c.2356C>T | nonsense | pathogenic |
| T14 | — | — | — | — |
| T15 | TSC2(Exon10) | c.853T>C | missense | likely pathogenic |
| T16 | TSC2(Exon14) | c.1435del T | frameshift | pathogenic |
| T17 | TSC2(Exon9) | c.826_827del AT | frameshift | pathogenic |
| T18 | TSC2(Exon2) | c.135_136del GA | frameshift | pathogenic |
| T19 | TSC1(Exon9) | c.892_893insC | frameshift | pathogenic |
| T20 | TSC2(Exon34) | c.4113_4114 insG | frameshift | pathogenic |
| T21 | TSC2(Exon10) | c.880G>T | nonsense | pathogenic |
| T22 | TSC2(Intron18) | c.1946+1G>A | splice site variant | likely pathogenic |
| T23 | TSC2(Exon41) | c.5384_5385delGC | frameshift | pathogenic |
| T24 | TSC2(Exon36) | c.4630A>T | nonsense | pathogenic |
| T25 | TSC2(Exon14) | c.1372C>T | nonsense | pathogenic |
| T26 | TSC2(Exon41) | c.5228G>A | missense | likely pathogenic |
| T27 | TSC2(Exon41) | c.5227C>T | missense | likely pathogenic |
| T28 | TSC2(Exon24) | c.2713C>T | missense | likely pathogenic |
| T29 | TSC2(Exon38) | c.4858C>T | missense | likely pathogenic |
| T30 | TSC2(Exon19) | c.2032G>A | missense | benign |
| T31 | TSC2(Exon41) | c.5228G>A | missense | likely pathogenic |
| T32 | TSC1(Exon18) | c.2227C>T | nonsense | pathogenic |
| T33 | TSC2(Exon16) | c.1700T>C | missense | uncertain significance |
| T34 | TSC1(Exon8) | c.733C>T | nonsense | pathogenic |
| T35 | TSC2(Intron10) | c.976-15G>A | splice | likely pathogenic |
| T36 | TSC2(Exon11) | c.1073G>A | nonsense | likely pathogenic |
| T37 | TSC2(Exon36) | c.4589C>A | nonsense | pathogenic |
| T38 | TSC2(Exon22) | c.2538delC | frameshift | pathogenic |
| T39 | TSC2(Exon30) | c.3412C>T | nonsense | pathogenic |
| T40 | TSC2(Exon41) | c.5227_5244delCGGCTC  CGCCACATCAAG | deletion | likely pathogenic |
| T41 | TSC2(Exon38) | c.4952A>G | missense | likely pathogenic |
| T42 | — | — | — | — |
| T43 | TSC2(Exon41)  TSC2(Exon3-10) | c.5234G>A  Loss of heterozygosity | missense  CNV | uncertain significance  pathogenic |
| T44 | TSC2(Exon16-22) | Loss of heterozygosity | CNV | pathogenic |
| T45 | — | — | — | — |
| T46 | — | — | — | — |
| T47 | — | — | — | — |
| T48 | — | — | — | — |
| T49 | TSC2(Exon32) | c.4054C>T | nonsense | likely pathogenic |
| T50 | TSC2(Exon34-42) | Loss of heterozygosity | CNV | likely pathogenic |
| T51 | TSC1(Exon17) | c.2119C>T | nonsense | likely pathogenic |
| T52 | TSC2(Exon37) | c.4728del G | frameshift | likely pathogenic |
| T53 | — | — | — | — |
| T54 | TSC2(Exon12) | c.1139T>C | missense | uncertain significance |
| T55 | TSC1(Exon14) | c.1708_1709del | frameshift | pathogenic |
| T56 | TSC2(Exon18) | c.1864C>T | nonsense | pathogenic |
| T57 | TSC2(Exon30) | c.3412C>T | nonsense | pathogenic |
| T58 | TSC2(Exon3) | c.182_183insA | frameshift | pathogenic |
| T59 | TSC2(Exon11) | c.1064_1065insG | frameshift | pathogenic |
| T60 | TSC2(Exon15) | c.1488delC* | frameshift | likely pathogenic* |
| T61 | — | — | — | — |
| T62 | TSC2(Exon37) | c.4905C>A | nonsense | likely pathogenic |
| T63 | — | — | — | — |
| T64 | TSC2(Exon17) | c.1789C>T | missense | likely pathogenic |
| T65 | — | — | — | — |
| T66 | TSC2(Exon15) | c.1513C>T | nonsense | pathogenic |
| T67 | TSC2(Exon24) | c.2644delA | frameshift | likely pathogenic |
| T68 | TSC1(Exon9) | c.812_813del | frameshift | pathogenic |
| T69 | TSC2(Exon20) | c.2295dupC | frameshift | pathogenic |
| T70 | TSC2(Exon1-20) | Loss of heterozygosity | CNV | pathogenic |
| T71 | TSC1(Exon15) | c.1690G>T | nonsense | likely pathogenic |
| T72 | TSC2(Exon41) | c.5228G>A | missense | likely pathogenic |
| T73 | TSC2(Exon4-10) | Loss of heterozygosity | CNV | pathogenic |
| T74 | TSC1(Exon10) | c.989dupT | frameshift | pathogenic |
| T75 | — | — | — | — |
| T76 | TSC2(Exon34) | c.4469A>G | missense | uncertain significance |
| T77 | TSC2(Exon10) | c.910_911insG | frameshift | pathogenic |
| T78 | — | — | — | — |
| T79 | TSC2(Exon22) | c.2365G>A | missense | benign |
| T80 | — | — | — | — |
| T81 | TSC2(Exon20) | c.2098-2A>G | splice | likely pathogenic |
| T82 | TSC2(Exon16) | c.1716G>T | missense | likely pathogenic |
| T84 | TSC2(Exon9) | c.826_827del | frameshift | pathogenic |
| T85 | — | — | — | — |
| T88 | TSC2(Intron10) | c.976-15G>A | splice | pathogenic |
| T92 | TSC2(Exon23) | c.2552C>A | missense | uncertain significance |
| T93 | TSC1(Exon14) | Loss of heterozygosity | CNV | likely pathogenic |
| T95 | TSC2(Exon14) | c.1385G>A | missense | likely pathogenic |
| T96 | TSC2(Exon29) | c.3310C>T | nonsense | pathogenic |
| T98 | TSC2(Exon24) | c. 2678T>G | missense | likely pathogenic |
| T99 | TSC2(Exon25) | c.2735T>C | missense | uncertain significance |
| T100 | TSC2(Exon34) | c.4013C>A | nonsense | likely pathogenic |
| T101 | TSC2 (Exon6) | c.557delT | frameshift | pathogenic |
| T102 | TSC1(Exon20) | c. 2623C>T | nonsense | pathogenic |
| T104 | TSC2(Exon39) | c.5027G>A | missense | uncertain significance |
| T105 | TSC2(Exon11) | c.1108C>T | nonsense | likely pathogenic |
| T106 | TSC2(Exon39) | c.5030T>C | missense | uncertain significance |
| T107 | TSC2(Exon20) | c.2125G>T | missense | uncertain significance |
| T108 | TSC2(Exon41) | c.5228G>T | missense | uncertain significance |
| T109 | TSC1(Exon7) | c.682C>T | nonsense | pathogenic |
| T110 | TSC1(Exon16) | c.1960C>G | missense | uncertain significance |
| T111 | TSC2(Exon22) | c.2447C>T | missense | uncertain significance |
| T112 | TSC2(Exon37) | c.4813C>T | nonsense | pathogenic |

* The mutation rate at this site in patients is only 11%, which is considered mosaicism.

**CNV**, copy number variant

**Reference:**

Richards S, Aziz N, Bale S, et al. Standards and guidelines for the interpretation of sequence variants: a joint consensus recommendation of the American College of Medical Genetics and Genomics and the Association for Molecular Pathology. Genet Med 2015;17:405-424. doi: 10.1038/gim.2015.30
